# Alphacoronaviruses Are Common in Bats in the Upper Midwestern United States

**DOI:** 10.3390/v14020184

**Published:** 2022-01-19

**Authors:** Reagan Schaeffer, Gun Temeeyasen, Ben M. Hause

**Affiliations:** Department of Veterinary and Biomedical Sciences, South Dakota State University, Brookings, SD 57007, USA; reagan.schaeffer@jacks.sdstate.edu (R.S.); gun.temeeyasen@sdstate.edu (G.T.)

**Keywords:** *Alphacoronavirus*, coronavirus, *Eptesicus*, bats, zoonotic

## Abstract

Bats are a reservoir for coronaviruses (CoVs) that periodically spill over to humans, as evidenced by severe acute respiratory syndrome coronavirus (SARS-CoV) and SARS-CoV-2. A collection of 174 bat samples originating from South Dakota, Minnesota, Iowa, and Nebraska submitted for rabies virus testing due to human exposure were analyzed using a pan-coronavirus PCR. A previously partially characterized CoV, *Eptesicus* bat CoV, was identified in 12 (6.9%) samples by nested RT-PCR. Six near-complete genomes were determined. Genetic analysis found a high similarity between all CoV-positive samples, Rocky Mountain bat CoV 65 and alphacoronavirus HCQD-2020 recently identified in South Korea. Phylogenetic analysis of genome sequences showed EbCoV is closely related to bat CoV HKU2 and swine acute diarrhea syndrome CoV; however, topological incongruences were noted for the spike gene that was more closely related to porcine epidemic diarrhea virus. Similar to some alphaCoVs, a novel gene, ORF7, was discovered downstream of the nucleocapsid, whose protein lacked similarity to known proteins. The widespread circulation of EbCoV with similarities to bat viruses that have spilled over to swine warrants further surveillance.

## 1. Introduction

Coronaviruses (CoVs) are enveloped, positive-sense RNA viruses in the family *Coronaviridae*, of the order *Nidovirales* [1]. CoVs belong to the realm *Riboviria*, a taxon of viruses that use RNA-dependent RNA-polymerases (RdRps) for reverse transcription of the viral genome, which replicates in the cytoplasm of the host cell [1]. CoVs comprise four genera from the *Coronavirinae* subfamily: *Alphacoronavirus*, *Betacoronavirus*, *Gammacoronavirus*, and *Deltacoronavirus* [1]. Alphacoronaviruses (alphaCoVs) and betacoronaviruses (betaCoVs) infect a variety of mammalian species, including humans, cows, swine, rodents, cats, dogs, and bats, while deltacoronaviruses and gammacoronaviruses principally infect avian species [2,3,4].

Coronaviruses are the largest known positive-sense RNA viruses, with genome sizes of approximately 27–33 kilobases [3]. The approximate 5′-two thirds of the genome encode a large polyprotein that is post-translationally cleaved into a variety of non-structural proteins necessary for viral replication and antagonism of the host immune response. A propensity for recombination allows for rapid virus mutation and host switching. The spike (S) glycoprotein projects from the virion surface and mediates viral entry into host cells [5,6]. Consequently, the S protein is a critical determinant of viral host range and tissue tropism [6]. The remaining structural proteins include the membrane (M), nucleocapsid (N), and envelope (E) proteins. Accessory genes are also variably encoded in the CoV genome.

Prior to the discovery of severe acute respiratory syndrome coronavirus (SARS-CoV) in 2003, CoVs were not considered highly pathogenic in humans, with infection typically resulting in mild respiratory disease [7,8]. In contrast, the past 20 years have seen three worldwide pandemics caused by suspected bat-origin CoVs with devastating effects on human health. To date, seven CoVs are known to cause acute respiratory tract infections in humans [7,9,10]. Besides SARS-CoV and SARS-CoV-2, five other human pathogenic CoVs have been identified. Human CoV-229E (HCoV-229E) and human CoV-OC43 (HCoV-OC43) were discovered in the 1960s [7,11]. Human CoV-NL63 (HCoV-NL63) and human CoV-HKU1 (HCoV-HKU1) were discovered in 2004 and 2005, respectively [8]. In November 2012, Middle East Respiratory Syndrome coronavirus (MERS-CoV) was identified [12]. While four of the known coronaviruses infecting humans, HCoV-229E, HCoV-OC43, HCoV-NL63, and HCoV-HKU1, cause mild respiratory symptoms, the deadly outbreaks of MERS-CoV, SARS-CoV, and SARS-CoV-2 demonstrate the need for coronavirus surveillance [11].

Several human CoVs share ancestry with bat CoVs, with spillover from closely related bat CoVs suspected for some human CoVs such as SARS-CoV [13]. It is thought that SARS-CoV-2 also originated bats, although more research is needed to verify this [14]. In addition, previous research has shown that bats are reservoirs for diverse alphaCoVs and betaCoVs [4].

Compared to elsewhere in the world, comparably little surveillance for bat CoVs have been performed in the United States. A pan-coronavirus PCR-based study of seven bat species collected from four sites in the Rocky Mountain region identified three distinct clusters of alpha-CoVs based on sequencing of a 440 nt region of the RdRp domain [15]. One of these CoVs, Rocky Mountain bat CoV 65, was identified from *Eptesicus fuscus*, while the remaining CoV-positive bats belonged to the genera *Myotis*. Similarly, metagenomic analysis of *E.* *fuscus*, *M. lucifugus*, and *Perimyotis subflavus* bats from the Eastern U.S. identified alphaCoV sequences originating from at least three viruses [16]. More broadly, a review of bat CoVs detected in the Americas found that 89.4% of the 151 CoVs described belonging to *Alphacoronavirus*, with the remainder in *Betacoronavirus* [17].

Here, to identify potentially novel bat CoVs that may pose a risk to humans, we screened bats submitted for rabies testing due to human exposure using a pan-coronavirus PCR. Six complete and six partial genomes were assembled that were highly similar to the partial genome of Rocky Mountain bat coronavirus 65. Phylogenetic analyses revealed evolutionary relationships to bat, livestock, and companion animal CoVs.

## 2. Materials and Methods

### 2.1. Ethics Statement

Bat samples were submitted to the South Dakota State University Animal Disease Research and Diagnostic Laboratory (SDSU ADRDL) for rabies testing due to human exposure, and as such, no specific approval was required. All tissue processing, RNA extraction, and viral isolation of the bat samples were performed in the SDSU ADRDL BSL-3 laboratory.

### 2.2. Sample Collection and Processing

Big brown bats (*Eptesicus fuscus*) submitted to SDSU ADRDL for rabies testing from March 2020 to July 2021 were used in this study. All 174 bat samples tested negative for rabies virus by fluorescent antibody detection. The bats originated from South Dakota, Minnesota, Iowa, and Nebraska. Tissue homogenates were assembled from pooled viscera in phosphate-buffered saline and clarified by centrifugation at 14,000× *g* for five minutes.

### 2.3. RNA Extractions

RNA was extracted from the bat homogenates using a Kingfisher sample purification system combined with MagMAX viral RNA extraction reagents.

### 2.4. Nested Pan-CoV PCR

A nested pan-CoV PCR was performed to detect CoV-positive samples. The outer PCR targeting the RdRp domain was adapted from a previously described assay [18]. An amount of 2 µL extracted RNA, 5 µL QIAGEN One Step RT-PCR 5X Buffer, 1 µL dNTPs (QIAGEN, 10 mM each), 1 µL enzyme (QIAGEN), 2.5 µL 10 µM panCoV-F2 (5′-AARTTYTAYGGHGGYTGG-3′), 2.5 µL 10 µM panCoV-R1 (5′-GARCARAATTCATGHGGDCC-3′), 0.25 µL 40 U/µL RNaseOut (Invitrogen), and enough water to achieve a total of 25 µL were combined. Using the Applied Biosystems 2720 Thermal Cycler, reverse transcription and initial denaturation were performed for 30 min at 50 °C and 15 min at 95 °C, followed by 35 cycles of 40 s at 94 °C, 40 s at 52 °C, and 1 min at 72 °C, with a final extension for 10 min at 72 °C, and a holding temperature of 4 °C. The products were visualized on a 1.5% agarose gel, with an expected band size of ~600 bp.

The nested PCR was adapted from a previously developed assay [19]. The outer PCR product was diluted at 1:10. One microliter of the diluted outer PCR product was added to a Master Mix containing 0.25 µL TaKaRa rTaq (5 U/µL), 5 µL 10X PCR buffer (TaKaRa), 4 µL dNTPs (2.5 mM each), 2 µL 10 µM Pan_CoV_F-3 (5′-GAYTAYCCHAARTGTGAYMGH-3′), 2 µL 10 µM Pan_CoV_R-1 (5′-CCRTCATCAGAHARWATCAT-3′), and water to bring the amount to 50 µL. Thermal cycling was performed using the Applied Biosystems 2720 Thermal Cycler with 40 cycles of 30 s at 94 °C, 30 s at 48 °C, and 1 min at 72 °C, with a hold of 4 °C. The products were visualized on a 1.5% agarose gel, with an expected band size of ~430 bp.

The detection breadth of the nested PCR assay was assessed using 15 CoV references derived from cell culture. Reference viruses included porcine epidemic diarrhea virus (PEDV), five strains of bovine coronavirus (BCV), porcine deltacoronavirus (PDCoV), canine respiratory coronavirus (CRCoV), HuCoV-OC43, three strains of canine coronavirus (CCoV), two strains of feline coronavirus (FCoV), and equine coronavirus.

### 2.5. Sanger Sequencing

Nested PCR amplicons were purified with the NEB Monarch PCR and DNA Cleanup Kit, following manufacturer instructions for DNA cleanup and concentration. The concentration of the purified CoV samples was measured with the Invitrogen Qubit dsDNA High Sensitivity (HS) Assay Kit. Samples were diluted to a concentration of 4 ng/µL and submitted to the SDSU ADRDL for Sanger sequencing of the RdRp gene to confirm CoV PCR results.

### 2.6. Metagenomic Sequencing

Following the digestion of unprotected nucleic acids by a cocktail of DNases and RNases, RNA was extracted from the CoV-positive homogenates using the QIAamp viral RNA kit (Qiagen, Hilden, Germany). For samples that yielded incomplete genome sequences after the initial sequencing attempt, nuclease digested RNA was further purified using oligo-dT beads to enrich for polyadenylated RNA before continuing with sample preparation. Reverse transcription and second-strand synthesis were carried out using the SuperScript III First-Strand Synthesis System (Invitrogen) and Sequenase 2.0 DNA Polymerase (Applied Biosystems) using barcoded random hexamers FR26RV-N [20]. Complementary DNAs (cDNA) were then amplified by using TaKaRa rTaq with barcode primers FR20RV [20]. One ng of the resulting amplicons from each sample was used for library preparation. A Nextera XT library preparation kit was used to construct sequencing libraries, which were sequenced with an Illumina MiSeq machine. Using QIAGEN CLC Genomics, contigs were assembled de novo. Contigs were identified by BLASTX using the cloudblast feature implemented in Omicsbox.

### 2.7. Phylogenetic Analysis

Phylogenetic analysis was performed for genome sequences and for each gene individually, including species in the genera *Alphacoronavirus*, with SARS-CoV-2 included as an outgroup representing *Betacoronavirus*. Nucleotide sequences were aligned in MEGAX using ClustalW [21,22]. Phylogeny was inferred using the Maximum Likelihood method using the GTR+G model in MEGAX [21,22]. Tree topologies were assessed with 500 bootstrap replicates for genome and ORF1ab sequences, with 1000 bootstrap replicates utilized for remaining genes.

### 2.8. Recombination Analysis

Recombination analysis was performed on the full genome using SimPlot [23]. This was verified using RDP4 [24]. The window size was set at 400 and the step size at 200 for both analyses. Recombination analysis was performed for EbCoV and reference sequences for PEDV, HCoV-NL63, HCoV-229E, bat CoV strain HKU2, Bat CoV strain 1A, CCoV, feline infectious peritonitis virus (FIPV), and transmissible gastroenteritis virus (TGEV).

### 2.9. Viral Isolation

Viral isolation was attempted for positive CoV samples on Vero76 and immortalized *Eptesicus fuscus* kidney (Efk3) cells. Cells were grown at 37 °C with 5% CO_2_ in warmed DMEM media with 10% fetal bovine serum and 1% antibiotics and antimycotic (Gibco). Confluent monolayers in 12-well plates were inoculated with 100 µL of unfiltered tissue homogenate and 100 µL 0.22 µM filtered homogenate, respectively. Virus isolation was attempted using both growth media as well as DMEM containing trypsin. The cells were incubated for seven days and then passed to new confluent monolayers. After seven days, 140 µL of the cell culture was tested using the outer PCR method described in Section 2.4 after extracting RNA with the QIAamp viral RNA kit (Qiagen, Hilden, Germany). All cell cultures, filtered and unfiltered, were considered negative when no cytopathic effects were visible, and the outer PCR was negative according to gel electrophoresis.

### 2.10. Accession Numbers

The six complete bat CoV genomes were deposited into GenBank, according to the following accession numbers OL410607-OL410610, OL415261, and OL415262.

## 3. Results

### 3.1. The Pan-Coronavirus PCR Detected All 15 Reference Coronaviruses

**Figure 1:**
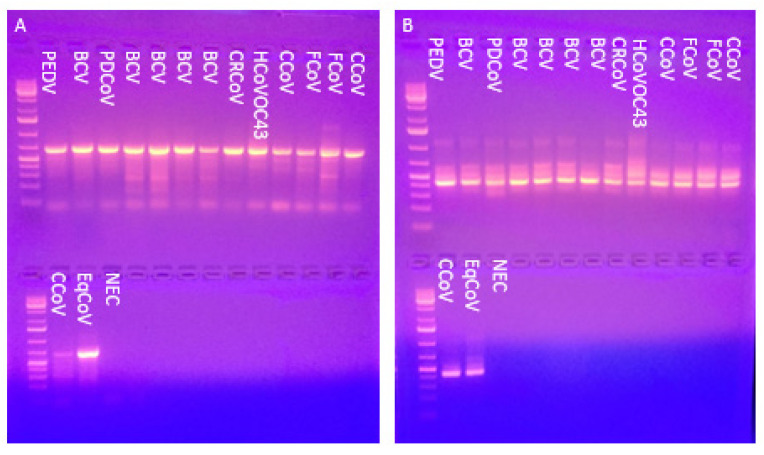
(**A**) Outer PCR for CoV references yielded a band ~600 bp. (**B**) Nested PCR for CoV references yielded a band ~430 bp. CoV references include porcine epidemic diarrhea virus (PEDV), five strains of bovine coronavirus (BCV), porcine deltacoronavirus (PDCoV), canine respiratory coronavirus (CRCoV), human coronavirus OC-43 (HuCoV-OC43), three strains of canine coronavirus (CCoV), two strains of feline coronavirus (FCoV), and equine coronavirus. NEC, negative extraction control.

The nested PCR sensitivity was tested with 15 known CoV references. All 15 viruses gave a single, intense band of the proper size for both outer and nested PCR reactions (Figure 1).

### 3.2. Novel Alphacoronavirus Discovered in Bats from South Dakota and Minnesota

Of 174 bat specimens, 12 homogenates (6.9%) were positive for CoV by nested PCR. The geographical distribution of samples and CoV-positives are shown in Table 1 and Supplementary Figure S1. A majority of both samples and CoV-positive samples originated in Minnehaha county, which is the most populous county in South Dakota (SD). Positive samples were approximately evenly distributed between urban (Minnehaha county; 7 out of 112 (6.3%)) and rural (all other counties; 5 out of 62 (8.1%)) sample sources. More samples were tested from the Eastern half of SD than the Western half.

Complete or near-complete coding regions of six genomes were assembled de novo. For the remaining positive samples, only partial RdRp domain sequences were determined. Genome completeness was assessed by open reading frame (ORF) and BLASTP analysis. The assembled genomes were 27,994 to 28,546 nucleotides in length and contained incomplete noncoding regions.

Sanger sequencing was performed on the nested PCR product for all 12 bat CoV positives. The Sanger sequences of the 12 positive bat CoV RdRp regions were analyzed by BLASTN, which found that the sequences were 96.0–98.2% similar to partial alphacoronavirus sequences determined from *Eptesicus fuscus* (Accession number JX537914.1). The previously determined partial *E. fuscus* CoV sequences were the basis for the inclusion of *Eptesicus* bat coronavirus (EbCoV) as a possible but not approved species in the genus *Alphacoronavirus* (https://ictv.global/taxonomy; accessed on 18 January 2022). Given the high similarity of our sequences to EbCoV, the six complete and six RdRp sequences determined here likely belong to the proposed species EbCoV.

### 3.3. Genome Features of Eptesicus Bat Coronavirus

**Figure 2:**
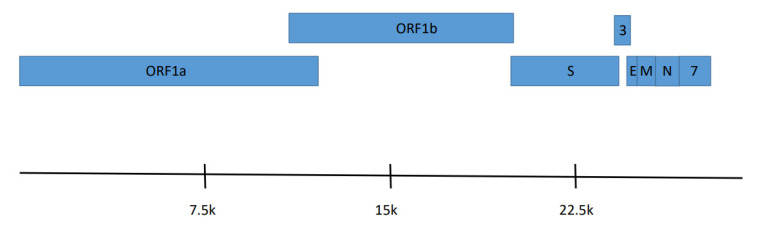
Genome organization of Eptesicus bat coronavirus. Genes for ORF1ab, Spike (S), ORF3 (3), Envelope (E), Membrane (M), Nucleocapsid (N), and ORF7 (7) are denoted.

The six EbCoV genomes shared 98–99% nucleotide identity with each other. Open reading frame and BLASTP analysis identified genes that encode the replicase polyproteins ORF1ab, S glycoprotein, envelope (E) protein, membrane (M) protein, and nucleocapsid (N) protein (Figure 2). A putative accessory gene ORF3 was located between S and E. Another putative accessory gene, ORF7, was located downstream of the N gene. All EbCoV proteins were most similar to alphacoronavirus HCQD-2020 (HCQD-2020) recently reported from South Korea [25]. Greater than 97% identity was found between EbCoV and HCQD-2020 for all proteins except S, where only 83.6% identity was found. The genes for S, ORF3, and E overlapped ORF1ab, S, and ORF3, respectively, by four, four, and 29 nucleotides (Table 2).

Post-translational cleavage of the replicase polyproteins into the complement of non-structural proteins was predicted by zCurve for the six EbCoV genomes (Supplementary Table S1) [26]. The cleavage sites for the non-structural proteins are shown in Supplementary Table S2 [26].

### 3.4. EbCoV Proteins Are Most Similar to Bat and Porcine Alphacoronaviruses

BLASTP analysis of EbCoV, using strain 14300 as a representative sequence, was performed to identify the closest homologs apart from HCQD-2020. The ORF1ab protein was most similar to bat alpha-CoVs identified in Asia and Europe, as well the porcine severe acute diarrhea syndrome coronavirus (SADS-CoV) and PEDV, all with approximately 60% identity. The S protein was most similar (47% identity) to NL63-related and 229E-related bat CoVs and PEDV. ORF3 had approximately 40% identity to homologs found in bat alpha-CoVs. The envelope (E) protein was 50% identical to bat and human alpha-CoVs. The membrane (M) protein was 65% identical to those found in bat and swine alpha-CoVs. The nucleocapsid (N) protein shared 79% identity to N from a partial sequence derived from the alpha-CoV *E. fuscus* Appalachian Ridge P1C837 and less than 50% similarity to other bat alpha-CoVs. For both nucleotide and amino acid sequences of the ORF7 gene, no significant similarities were found by BLASTN and BLASTP, respectively, apart from 97.1% identity to HCQD-2020.

### 3.5. Eptesicus Bat Coronavirus Is Most Closely Related to Bat CoV HKU2

**Figure 3:**
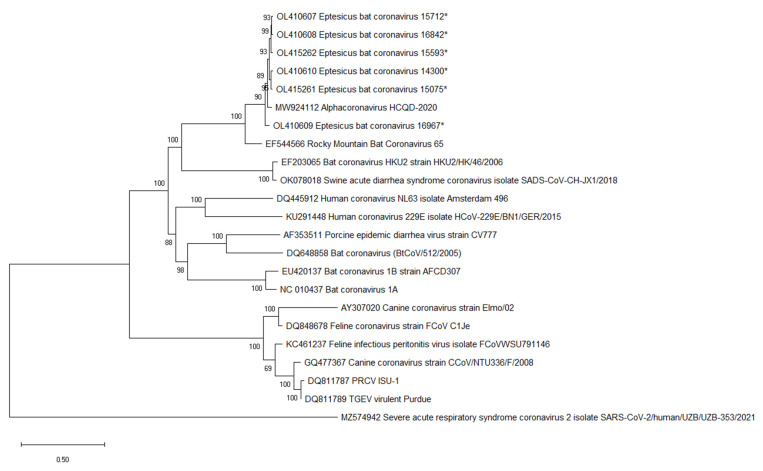
Phylogenetic analysis of complete genome sequences was performed in MegaX using the maximum likelihood method along with a GTR + G substitution model [21,22]. The bootstrap values were calculated from 500 replicates, and values greater than 70 are shown. Sequences determined here are indicated by *.

Phylogenetic trees were constructed using the genome nucleotide sequences for EbCoV and representative alpha-CoV (Figure 3). *Eptesicus* bat CoV formed a well-supported, monophyletic clade along with HCQD-2020 and Rocky Mountain bat CoV 65 (RMCoV65). The high, greater than 96%, nucleotide identity in the conserved replicase-encoding regions of the genome, along with close evolutionary histories, suggests that EbCoV, HCQD-2020, and RMCoV65 are members of the same species, EbCoV. The EbCoV clade was most closely related to a clade comprised of bat CoV HKU2 and SADS-CoV that occupied a well-supported sister clade position in the tree.

**Figure 4:**
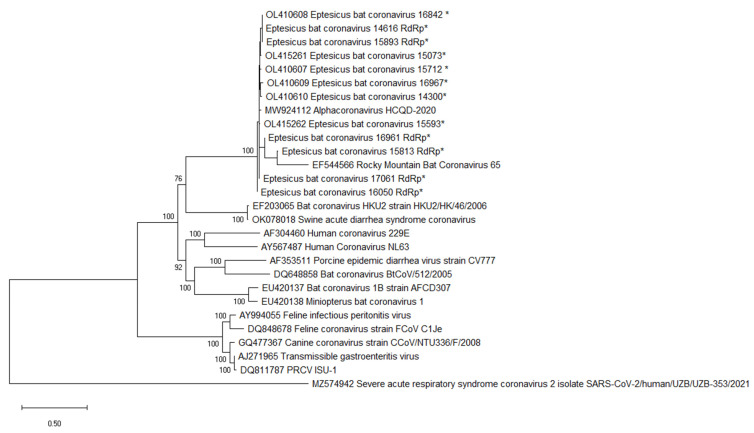
Phylogenetic analysis of ORF1ab nucleotide sequences was performed in MegaX using the maximum likelihood method along with a GTR + G substitution model [21,22]. The bootstrap values were calculated from 500 replicates, and values greater than 70 are shown. EbCoV sequences with the RdRp suffix are partial sequences derived from the nested PCR amplicon. Sequences determined here are indicated by *.

Phylogenetic analysis was performed on the ORF1ab gene derived from the near-complete genome sequences and partial RdRp sequences determined here. All strains formed a monophyletic clade that included RMCoV65 with strong bootstrap support. These results suggest that all 12 CoV-positive strains identified here are conspecific (Figure 4). Bat CoV strain HKU2 and SADS-CoV2 occupied a sister clade position to the EbCoV clade.

**Figure 5:**
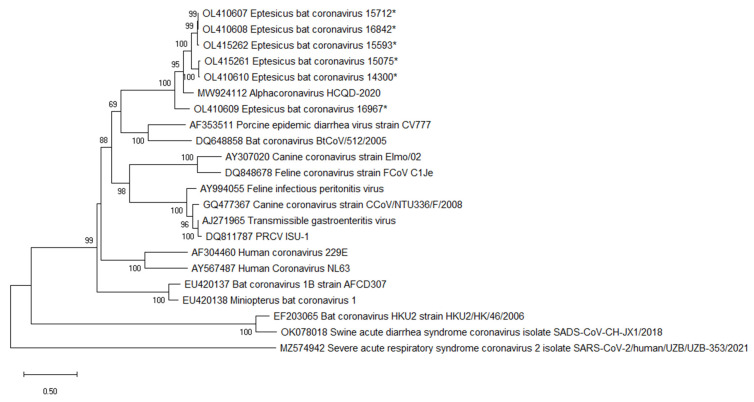
Spike gene sequence phylogenetic tree inferred by MegaX using maximum likelihood methodology and the GTR + G substitution model [21,22]. The bootstrap values are from 1000 replicates. Bootstrap values greater than 68 are shown. Sequences determined here are indicated by *.

Analysis of the S gene sequences likewise found a close evolutionary relationship between EbCoV strains from the U.S. and Korea that again formed a well-supported monophyletic lineage (Figure 5). A clade comprised of bat CoV strain 512 (BtCoV/512/2005) and PEDV formed a sister clade to the EbCoV with moderate bootstrap support. Interestingly, the EbCoV S gene sequences showed closer evolutionary relationships to swine and companion animals CoVs than the bat CoV HKU2 as seen for phylogenetic analysis of full genome sequences.

Phylogenetic analysis of the remaining genes found that all EbCoV strains formed a single, monophyletic clade (Supplementary Figures S2–S5). Tree topologies, however, lacked strong statistical support.

### 3.6. Recombination Analysis

Recombination analysis was performed for EbCoV and reference sequences for PEDV, HCoV-NL63, HCoV-229E, HKU2, Bat CoV strain 1A, CCoV, FIPV, and TGEV. No statistically significant (*p* < 0.05) recombination events were identified.

### 3.7. Virus Isolation

Attempts to isolate viruses from CoV-positive samples were unsuccessful on Vero76 and Efk3 cells. No cytopathic effects were evident, and all cell culture supernatants were negative for CoV by pan-CoV PCR following two passages.

## 4. Discussion

In this study, novel alpha-CoVs from *Eptesicus fuscus* in SD and Minnesota were characterized. The sequences of six genomes and six RdRp domains were determined directly from bats. The sequences were highly similar to an RdRp sequence previously determined from *E. fuscus* in the U.S., RMCoV65, which was proposed as a new species of alphaCoV, EbCoV. These sequences were additionally highly similar to a bat alpha-CoV identified from *E. serotinus* in South Korea while this manuscript was being prepared. Genetic and phylogenetic analyses demonstrated a close relationship between U.S. and Korean sequences for all genes. The high similarity between the *E. fuscus* derived CoV sequences here, the Korean CoV HCQD-2020 derived from *E. serotinus*, and the partial sequences previously recovered from *E. fuscus* in the U.S. suggests a single *Eptesicus* alpha-CoV species, EbCoV. Interestingly, *E. fuscus* and *E. serotinus* are closely related and may be conspecific [27].

EbCoV was identified in 12 of 174 (6.9%) of bats included in our study. These results demonstrate that EbCoV is common in *E. fuscus*. Importantly, the bats included in our study were all submitted for rabies virus testing due to human exposure. *E. fuscus* is common through the U.S. and is the dominant bat species in the upper Midwest. The insectivorous *E. fuscus* is synanthropic and frequently roosts in human-made structures, leading to frequent interaction with humans. Besides being a known reservoir for the rabies virus, *E. fuscus* harbors many viruses that may pose zoonotic risks [28,29,30,31,32].

In 2017, a severe outbreak of the enteric disease occurred in Chinese pig farms. A novel CoV, swine enteric alphacoronavirus, was isolated from diseased pigs. This virus, later renamed SADS-CoV, was found to be closely related to bat CoV HKU2 identified in *Rhinolophus* bats [33,34]. Phylogenetic analysis of EbCoV genome sequences here identified a close evolutionary relationship to SADS-CoV and HKU2 with strong bootstrap support. In addition, BLASTP analyses of EbCoV S proteins found PEDV S proteins were amongst the most similar known CoV proteins. Analysis of evolutionary signatures from EbCoV from a Korean bat found that animals in the order *Artiodactyla* were as likely to be infected with EbCoV as bats in the family *Vespertilionidae*, which include *Eptesicus* [25,35]. Together, these results suggest pigs may be susceptible to spillover from EbCoV.

To date, three alphaCoVs have been shown to infect pigs. Similar to SADS-CoV in China, the introduction of PEDV to the U.S. in 2013 led to an epidemic of severe disease [36]. While U.S. PEDV sequences were highly similar to contemporaneous Chinese PEDV, phylogenetic analyses support an evolutionary bat origin for PEDV.

Phylogenetic analysis of full CoV genome sequences found a well-supported sister clade relationship between HKU2 and EbCoV. Topological incongruences were evident, however, for single-gene phylogenies. For the spike gene, EbCoV was most closely related to a clade containing PEDV and only distantly related to HKU2. Despite this, recombination analysis failed to identify evidence of recombination. We hypothesize that the dearth of CoV sequences originating from wildlife in North America has prevented the characterization of close relatives to EbCoV that would allow for a better understanding of its evolutionary history.

Compared to elsewhere in the world, comparably little surveillance for bat CoVs have been performed in the Americas. The vast majority, 89%, of New World CoVs are alpha-CoVs, which includes all CoVs identified in *Eptesicus* [17]. Here we identified 12 CoV-positive *E. fuscus* using pan-CoV primers, all of which were EbCoV, suggesting little CoV diversity in *E.fuscus* in the upper Midwest. Experimental inoculation of *E. fuscus* with SARS-CoV-2 also found no evidence of infection [37]. As our study utilized samples collected from limited geography, further surveillance is needed to assess the breadth of CoV diversity in U.S. bats.

## Figures and Tables

**Table 1:** Distribution of bat samples tested and pan-coronavirus positive samples.

County and State	Samples	Positive
Minnehaha, SD	112	7
Yankton, SD	6	1
Charles Mix, SD	4	
Turner, SD	3	1
Clay, SD	1	
Brule, SD	1	
McCook, SD	1	
Lake, SD	5	
Hanson, SD	1	
Grant, SD	1	
Brookings, SD	5	
Codington, SD	3	1
Spink, SD	1	
Davison, SD	1	
Hutchinson, SD	4	
Marshall, SD	1	
Hughes, SD	1	
Beadle, SD	1	
Custer, SD	2	
Pennington, SD	2	
Hamlin, SD	1	
Tripp, SD	1	
Deuel, SD	1	1
Sanborn, SD	1	
Hanson, MN	1	
Lac qui Parle, MN	1	
Lyon, MN	4	
Rock, MN	2	1
Redwood, MN	1	
Pipestone, MN	1	
Sioux, IA	2	
Lyon, IA	1	
Boyd, NE	1	

**Table 2:** Genome organization of *Eptesicus* bat CoV strains determined from *E. fuscus*. Nucleotides for genes are shown. Predicted protein lengths are shown in parentheses. Incomplete sequences are indicated by *.

EbCoV Strain	ORF1ab	S	ORF3	E	M	N	ORF7
15,712	252–20,845 (6864)	20,842–24,873 (1343)	24,870–25,544 (224)	25,516–25,761 (81)	25,772–26,449 (225)	26,467–27,627 (386)	27,630–28,466 (278)
16,842	357–20,356 (6666) *	20,353–24,384 (1343)	24,381–25,055 (224)	25,027–25,272 (81)	25,283–25,960 (225)	25,978–27,138 (386)	27,141–27,977 (278)
16,964	97–20,690 (6864)	20,687–24,730 (1347)	24,727–25,401 (224)	25,373–25,618 (81)	25,629–26,306 (225)	26,324–27,484 (386)	27,487–28,323 (278)
14,300	366–20,362 (6665) *	20,359–24,408 (1349)	24,405–25,079 (224)	25,051–25,296 (81)	25,307–25,984 (225)	26,002–27,159 (385)	27,162–27,998 (278)
15,073	366–20,365 (6666) *	20,362–24,411 (1349)	24,408–25,082 (224)	25,054–25,299 (81)	25,310–25,987 (225)	26,005–27,165 (386)	27,168–28,004 (278)
15,593	306–20,905 (6866)	20,902–24,933 (1343)	24,930–25,604 (224)	25,513–25,821 (102)	25,832–26,509 (225)	26,527–27,687 (386)	27,690–28,526 (278)

## Data Availability

The six complete bat CoV genomes were deposited into GenBank, according to the following accession numbers OL410607-OL410610, OL415261, and OL415262.

## Supplementary Materials

The following are available online at https://www.mdpi.com/article/10.3390/v14020184/s1, Figure S1: Map of South Dakota counties showing distribution of bat samples included in this study. Color-coded counties represent number of bat samples; Figure S2: Phylogenetic analysis of the ORF3 gene sequences; Figure S3: Phylogenetic analysis of the envelope gene sequences; Figure S4: Phylogenetic analysis of the membrane gene sequences; Figure S5: Phylogenetic analysis of nucleocapsid gene sequences; Table S1: Predicted sizes for *Eptesicus* bat CoV non-structural proteins derived from polyprotein ORF1ab. *, incomplete sequence; Table S2: Predicted cleavage sites for the non-structural proteins (nsp) of *Eptesicus* bat coronavirus.
