# Supplementary material for: Alphacoronaviruses Are Common in Bats in the Upper Midwestern United States
Source: Viruses. 2022 Jan 19;14(2):184. doi: 10.3390/v14020184 (PMC8877427; doi:10.3390/v14020184)
Supplement: Supplementary file 1 [file viruses-14-00184-s001.zip › Tables S1 and S2.pdf]

Table S1. Predicted sizes for *Eptesicus* bat CoV non-structural proteins derived from polyprotein ORF1ab. *, incomplete sequence.

EbCoV Strain	Nsp1	Nsp2	Nsp3	Nsp4	Nsp5	Nsp6	Nsp7	Nsp8	Nsp9	Nsp10	Nsp11/12	Nsp13	Nsp14	Nsp15	Nsp16
15593	282	608	1704	479	302	279	83	195	109	135	927	597	519	347	300
15075	82*	608	1704	479	302	279	83	195	109	135	927	597	519	347	300
15712	282	608	1702	479	302	279	83	195	109	135	927	597	519	347	300
16842	82*	608	1704	479	302	279	83	195	109	135	927	597	519	347	300
16964	282	608	1702	479	302	279	83	195	109	135	927	597	519	347	300
14300	82*	608	1703	479	302	279	83	195	109	135	927	597	519	347	300

Table S2. Predicted cleavage sites for the non-structural proteins (nsp) of *Eptesicus* bat coronavirus

nsp	Cleavage site
nsp2	SSLQ|AGLR
nsp3	VTLQ|SGRK
nsp4	SSVQ|SKLT
nsp5	AMLQ|SIAS
nsp6	VKLQ|NNEV
nsp7	IRLQ|AGKQ
nsp9	ANVQ|SFDQ
nsp10	TVLQ|ASGM
nsp11	TDLQ|ATEG
nsp12	TKIQ|GLEN
nsp13	PQLQ|SAEW
