# Supplementary figures and images for: Alphacoronaviruses Are Common in Bats in the Upper Midwestern United States
Source: Viruses. 2022 Jan 19;14(2):184. doi: 10.3390/v14020184 (PMC8877427; doi:10.3390/v14020184)

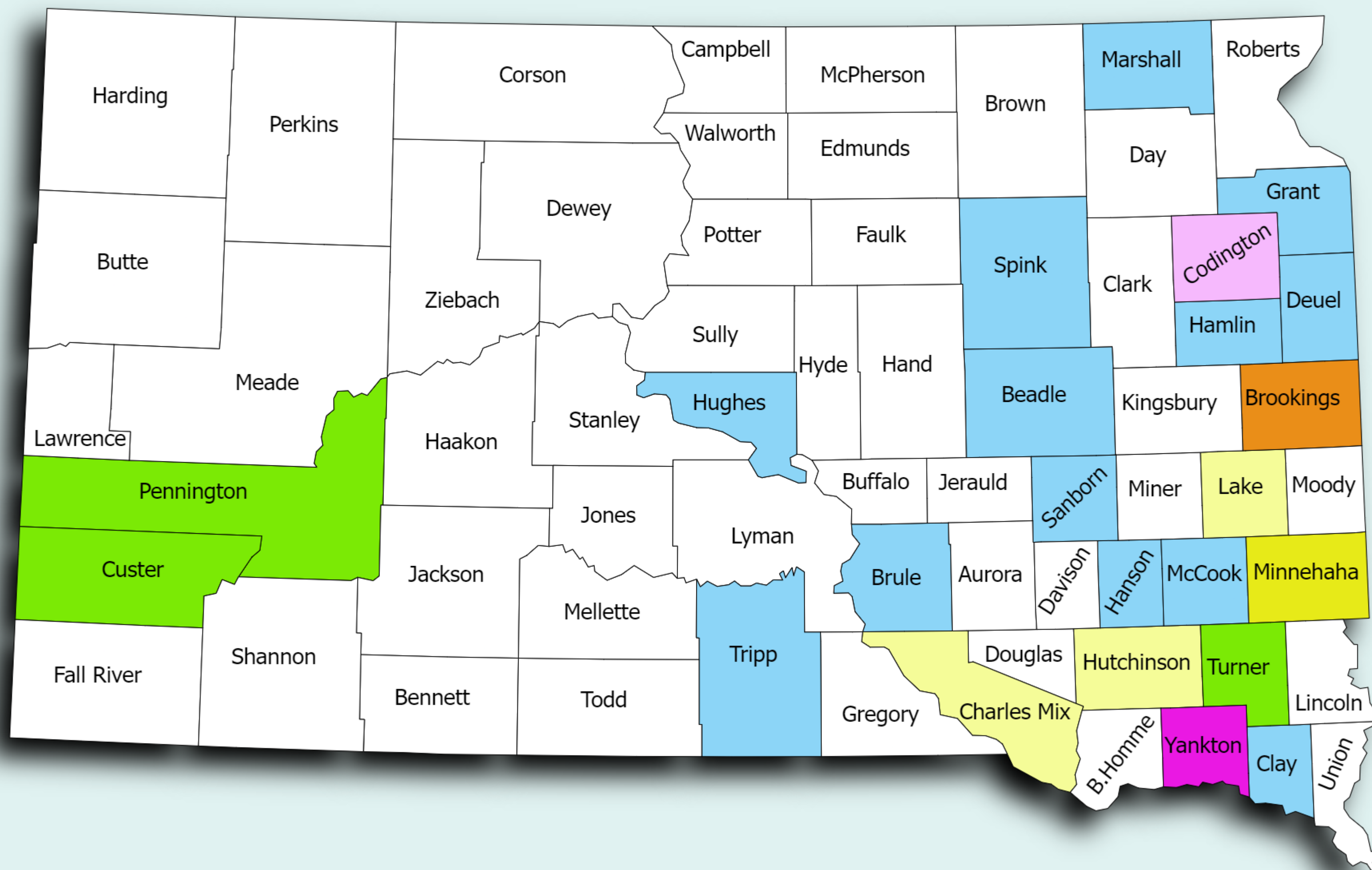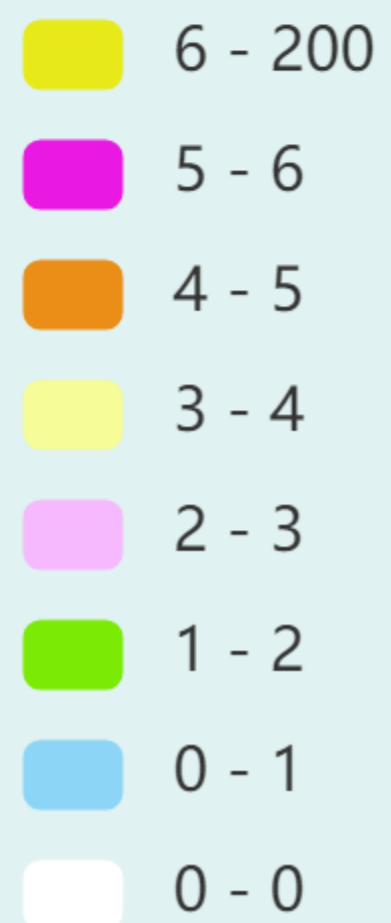

Supplement: Supplementary file 1 [file viruses-14-00184-s001.zip › Figure S1.pdf]

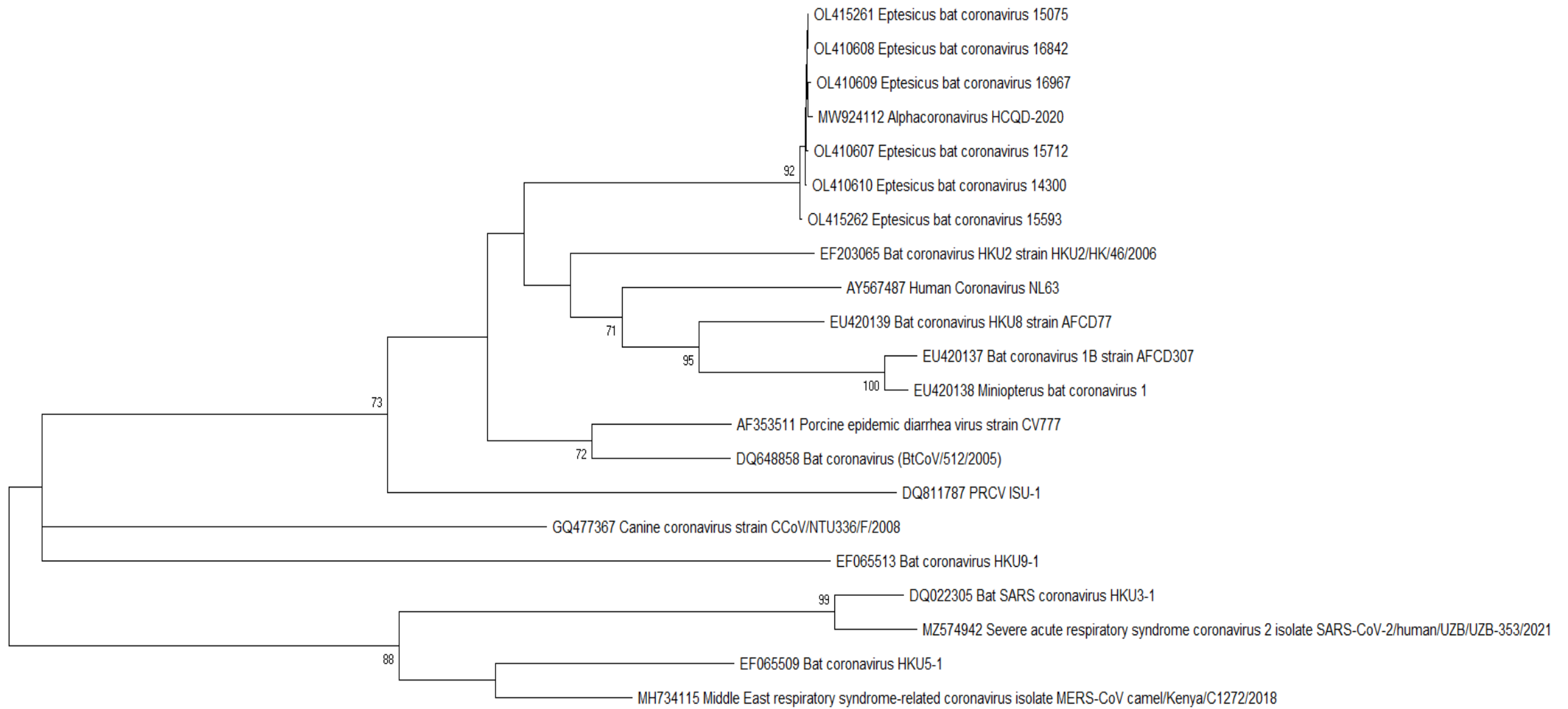

0.50

Supplement: Supplementary file 1 [file viruses-14-00184-s001.zip › Figure S2.pdf]

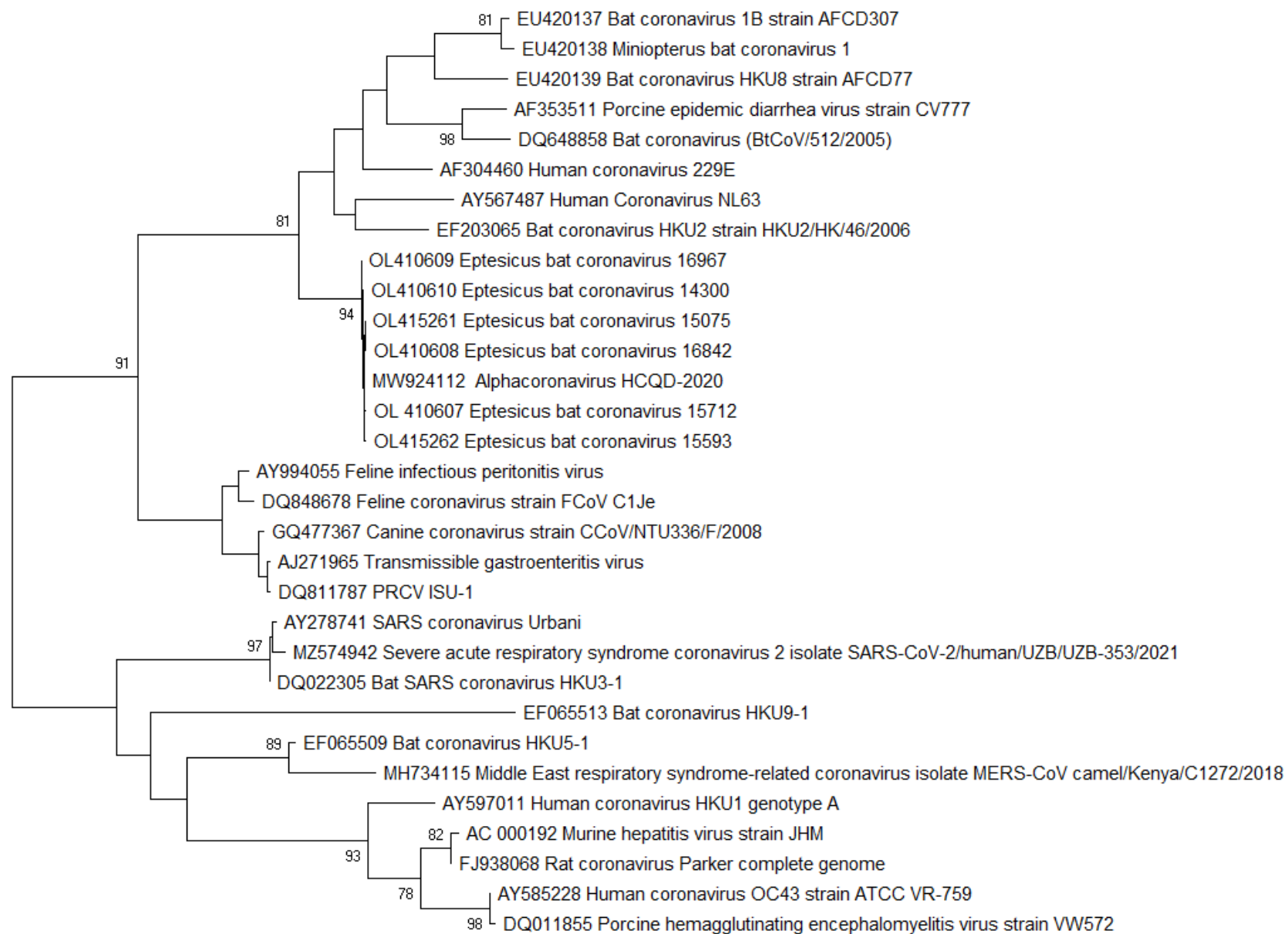

0.50

Supplement: Supplementary file 1 [file viruses-14-00184-s001.zip › Figure S3.pdf]

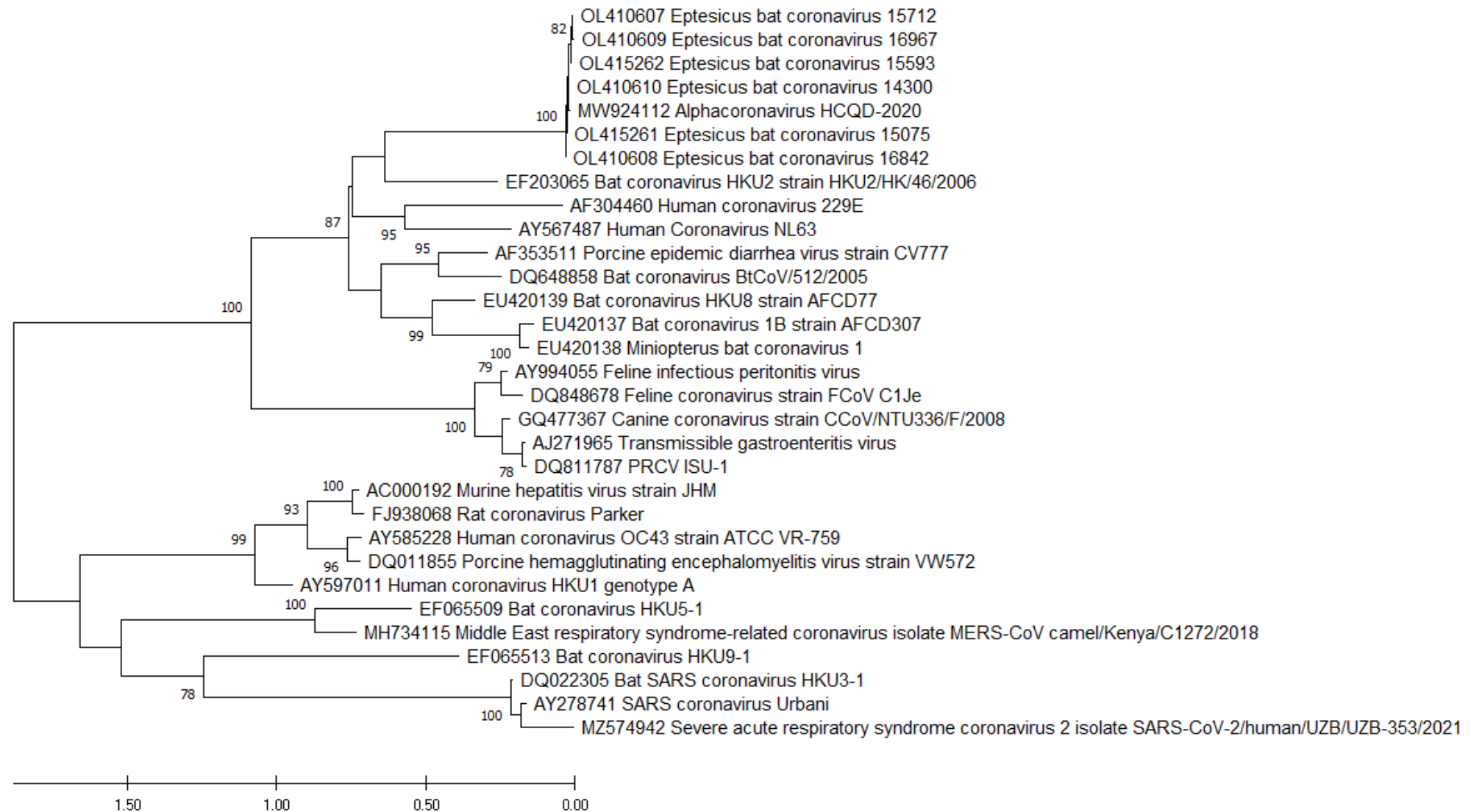

Supplement: Supplementary file 1 [file viruses-14-00184-s001.zip › Figure S4.pdf]

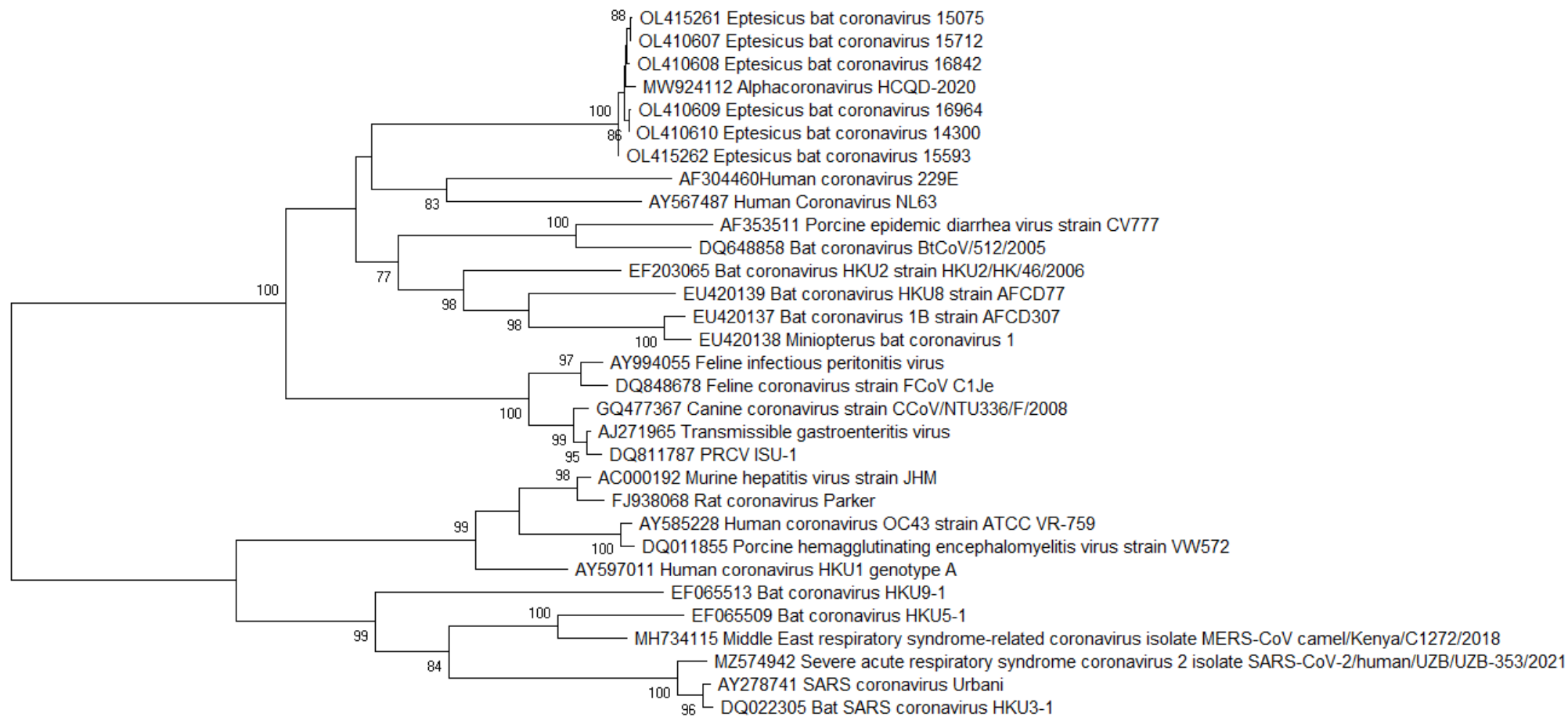

0.20

Supplement: Supplementary file 1 [file viruses-14-00184-s001.zip › Figure S5.pdf]
